# Impaired nitric oxide‐dependent endothelial function in young male individuals with obesity before the onset of symptoms and complications

**DOI:** 10.1113/EP093109

**Published:** 2025-11-22

**Authors:** Lucrezia Zuccarelli, Giovanni Baldassarre, Adele Bondesan, Diana Caroli, Roberta De Micheli, Gabriella Tringali, Thomas Favaretto, Joanna Suraj‐Prazmowska, Anna Kurpinska, Joanna Majerczak, Stefan Chlopicki, Jerzy A. Zoladz, Alessandro Sartorio, Bruno Grassi

**Affiliations:** ^1^ Department of Medicine University of Udine Udine Italy; ^2^ Experimental Laboratory for Auxo‐endocrinological Research Istituto Auxologico Italiano, IRCCS Piancavallo‐Verbania Italy; ^3^ Jagiellonian Center for Experimental Therapeutics (JCET) Jagiellonian University Krakow Poland; ^4^ Chair of Exercise Physiology and Muscle Bioenergetics, Faculty of Health Sciences Jagiellonian University Medical College Krakow Poland; ^5^ Department of Pharmacology Jagiellonian University Medical College Krakow Poland

**Keywords:** exercise tolerance, endothelial function, nitric oxide, obesity, PLM

## Abstract

Endothelial dysfunction drives obesity‐related complications. Doppler ultrasound measurement of blood flow during 1‐min passive leg movements (PLM) is a valuable non‐invasive tool for assessing endothelial function and nitric oxide (NO)‐mediated vasodilation. The objectives of this work were t o identify endothelial dysfunction biomarkers in young individuals with obesity (OB) using the PLM test; to evaluate the effects of a rehabilitation programme on these biomarkers; and to explore associations between PLM data, oxidative metabolism and blood biomarkers of microvascular impairment. Fifteen male OB (age 17 ± 4 years; body mass 121.4 ± 24.1 kg; body mass index 39.3 ± 7.5 kg m^−2^) were tested before (PRE) and after (POST) a 3‐week multidisciplinary body mass reduction programme. Fifteen age‐matched normal‐weight males (CTRL) underwent PRE measurements. Participants performed an incremental exercise, a PLM test and underwent blood biomarker analysis. Peak oxygen uptake and ventilatory thresholds (mL kg^−1^ min^−1^) were ∼40% lower in OB versus CTRL (*P *< 0.001) and improved by ∼8% in OB POST versus PRE (*P *< 0.05). Plasma nitrite concentration was lower in OB (0.18 ± 0.09 µmol L^−1^) versus CTRL (0.51 ± 0.49; *P *= 0.02). Baseline blood flow, normalized for appendicular muscle mass, was similar between the two groups, whereas peak blood flow, Δpeak (difference between peak and baseline) and the area under the blood flow versus time curve were significantly lower in OB PRE, with improvements in OB POST. Several blood biomarkers of endothelial barrier function and permeability differed in OB versus CTRL. The blunted PLM‐induced hyperaemic response and lower plasma nitrite levels indicate impaired endothelial function in young individuals with obesity, occurring before the onset of cardiovascular and metabolic complications.

## Abstract (highlights)

- **What is the central question of this study?**
  Can passive leg movement (PLM)‐induced hyperaemia and related biomarkers detect early endothelial dysfunction in young individuals with obesity, and are these parameters modifiable by a short‐term rehabilitation programme?
- **What is the main finding and its importance?**
  Young males with obesity exhibit impaired PLM‐induced hyperaemia and reduced plasma nitrite levels, indicating early endothelial dysfunction. A 3‐week multidisciplinary intervention partially improved vascular responses and oxidative metabolism. These findings support the use of PLM as a non‐invasive tool for early detection and monitoring of endothelial dysfunction, before cardiovascular or metabolic complications arise.

## 1 INTRODUCTION

Obesity is a multifactorial condition characterized by excessive adipose tissue accumulation, which significantly and negatively impacts various organ systems and functions. It represents a risk factor for severe diseases and complications as well as a significant and growing public health concern (Green et al., 2017). Endothelial dysfunction, a key feature of obesity, leads to reduced nitric oxide (NO) bioavailability, increased endothelin‐1 levels, impaired vasodilation, a pro‐inflammatory state and increased oxidative stress and damage (Giannini et al., 2008; Hotamisligil, 2006; Urbina et al., 2009; Virdis et al., 2019; Widlansky et al., 2003). These changes are pivotal in the pathogenesis of the metabolic and cardiovascular complications associated with obesity, such as insulin resistance, dyslipidaemia, hypertension, microvascular changes and atherosclerosis (Aggoun, 2007). According to some studies (Urbina et al., 2009; Wong et al., 2018) endothelial impairments represent a critical risk for cardiovascular and metabolic health, also in young patients with obesity, who often exhibit early signs of endothelial, microvascular and macrovascular dysfunction compared to their non‐obese counterparts. This issue, however, appears to be controversial. Other studies (Harrell et al., 2015; Limberg et al., 2022) observed indeed a preserved microvascular endothelial function and a preserved β‐adrenergic‐mediated vasodilation in skeletal muscles of young adults with diabetes. These authors concluded that ageing and/or greater disease duration may be necessary to uncover overt impairments of these variables.

Physical exercise and caloric restriction may yield significant benefits in improving endothelial function in obese individuals (Wycherley et al., 2008), particularly in addressing the early onset of cardiovascular, microvascular and metabolic dysfunction. Data on endothelial function following prolonged (2–4 months) periods of body mass loss and physical activity interventions are inconsistent, showing either improvements (Sciacqua et al., 2003; Watts et al., 2004; Wong et al., 2018) or no changes (Clifton et al., 2005). No data are available in young populations of patients exposed to shorter interventions.

Measurement of the blood flow increase in the common femoral artery, by Doppler ultrasound (duplex mode, with simultaneous collection of vessel image with B‐mode and blood velocity with pulse‐wave mode), during 1 min of passive leg movements (PLM) of one leg, has emerged as a valuable non‐invasive functional evaluation tool of endothelial function, accessible and relatively easy to perform and interpret (Gifford & Richardson, 2017). Impairments of the hyperaemic response during PLM have been mainly related to inadequate NO production by endothelial cells and inadequate NO bioavailability (Gifford & Richardson, 2017). The PLM approach has been utilized to identify endothelial impairments in different populations, such as young subjects exposed to bed rest (Zuccarelli et al., 2021), middle‐age subjects commuting by car (*vs* subjects commuting by bicycle (Ursella et al., 2025), elderly subjects (Dorff et al., 2024; Groot et al., 2016), heart failure patients (Francisco et al., 2021; Witman et al., 2015), patients with chronic respiratory diseases (Ives et al., 2020), sepsis (Nelson et al., 2016), systemic sclerosis (Clifton et al., 2018), peripheral artery disease (Mortensen et al., 2012), heart transplantation (Hayman et al., 2010) and spinal cord injury (Venturelli et al., 2017).

The aims of the present study were: (1) to identify, by the PLM test, biomarkers of endothelial dysfunction in young individuals with obesity, before the onset of clinical symptoms or complications of the disease; (2) to compare these biomarkers with systemic variables related to oxidative metabolism during exercise and exercise tolerance; (3) to evaluate the effects of a rehabilitation intervention (exercise, caloric restriction, psychological counselling) on these variables; and (4) to identify, in the search for pathogenic mechanisms, associations between the impairments identified by the PLM test and blood biomarkers of microvascular impairment (endothelial dysfunction, thrombotic risk, endothelial permeability, endothelial inflammation and vascular function).

## 2 METHODS

### 2.1 Ethical approval

The adult patients and both parents of adolescent patients gave their written informed consent after receiving a detailed explanation of the experimental procedures before the start of the study. All procedures were approved by the ethics committee of the Italian Institute for Auxology, Milan, Italy (ID number: 2021_02_23_06; reference code: 01C122; acronym: FUENVASOB). All procedures were in accordance with the recommendations outlined in the *Declaration of Helsinki* (World Medical Association, 2001).

### 2.2 Subjects

Thirty volunteers participated in this study. Fifteen young males with obesity (OB) (mean ± standard deviation: age, 17.1 ± 4.1 years; body mass (BM), 121.4 ± 24.1 kg; body mass index (BMI), 39.3 ± 7.5 kg m^−2^), Tanner stage 4–5 (i.e. late puberty), who were admitted as in‐patients (Division of Auxology, Italian Institute for Auxology, Piancavallo, Italy) for a multidisciplinary body mass reduction programme, and 15 age‐ and sex‐matched controls (CTRL) (age: 20.4 ± 6.7 years) were studied. Some general and anthropometric characteristics of the two study groups are given in Table 1. BMI was calculated as BM divided by height^2^.

**TABLE 1:** General characteristics of control participants (CTRL), obese patients (OB) before (PRE) and following (POST) standard interventions.

	CTRL	OB–PRE	*P*	OB–POST	*P*
Number of participants	15	15	—	15	—
Age (years)	20.4 ± 6.7	17.1 ± 4.1	0.12	17.1 ± 4.2	0.56
Height (m)	1.74 ± 0.08	1.76 ± 0.09	0.69	1.75 ± 0.09	0.33
BM (kg)	66.6 ± 11.3	121.4 ± 24.1	**<0.001**	116.6 ± 23.9	**<0.001**
BMI (kg m^−2^)	22.0 ± 4.4	39.3 ± 7.5	**<0.001**	37.8 ± 8.1	**<0.001**
FFM (% BM)	82.7 ± 10.0	55.7 ± 4.0	**<0.001**	57.8 ± 5.8	**0.002**
FFM (kg)	55.4 ± 7.7	67.0 ± 10.4	**0.002**	66.7 ± 11.0	0.61
FM (% BM)	17.0 ± 10.0	44.3 ± 4.0	**<0.001**	42.2 ± 5.1	**0.002**
FM (kg)	12.1 ± 8.8	54.4 ± 14.6	**<0.001**	49.9 ± 14.4	**<0.001**
ASMM (kg)	3.53 ± 0.56	5.27 ± 1.05	**<0.001**	5.09 ± 1.03	**<0.001**
Waist circumference (cm)	84.8 ± 12.3	123.0 ± 15.7	**<0.001**	119.0 ± 15.9	**<0.001**
Diastolic blood pressure (mmHg)	74 ± 6	80 ± 6	**0.03**	79 ± 6	0.68
Systolic blood pressure (mmHg)	120 ± 5	129 ± 13	**0.04**	127 ± 10	0.43

Data are means ± SD. *P*‐values related to differences between CTRL and OB‐PRE were checked by means of Student's unpaired *t*‐test; *P*‐values related to differences between OB‐PRE and OB‐POST were checked by means of Student's paired *t*‐test. Values shown in bold indicate statistical significance. ASMM, appendicular skeletal muscle mass; BM, body mass; BMI, body mass index; FFM, fat free mass; FM, fat mass.

Inclusion criteria were: (1) body mass index (BMI) > 97th centile (OB) and between the 25th and 75th centile (CTRL) for age and sex, using the Italian growth charts (Cacciari et al., 2006); (2) no involvement in structured physical activity programmes (regular activity more than 120 min/week) during the 8 months preceding the study; (3) absence of overt uncompensated diabetes; and (4) absence of signs or symptoms referable to any significant cardiovascular, respiratory or orthopaedic disease contraindicating or significantly interfering with the tests.

### 2.3 Three‐week multidisciplinary body mass reduction programme

The 3‐week multidisciplinary BM reduction programme was an in‐hospital metabolic rehabilitation intervention that included full‐time residency with overnight stays, an energy‐restricted diet, physical exercise and psychological counselling. The diet's energy content was calculated by subtracting approximately 500 kcal from the measurement of resting energy expenditure, assessed after an overnight fast using open‐circuit indirect computerized calorimetry. In terms of macronutrients, the diet contained approximately 21% of the calories from proteins, 53% from carbohydrates and 26% from lipids.

The physical exercise programme consisted of 5 days per week of training, including the following. (1) One hour of dynamic standing and floor exercise with arms and legs (i.e. squats, step‐ups, jump rope, lunges, push‐ups and torso twists), performed at moderate intensity (monitored through a portable heart rate (HR) monitor; Polar RS400SD, Polar Electro Oy, Kempele, Finland) and under the guidance of a therapist. The intensity of the aforementioned aerobic activities was set at an HR corresponding to 60–80% of the individual's maximal HR, estimated as 220 minus age (in years). (2) Either 20–30 min of cycle ergometer exercise at 60 W, or 3–4 km outdoor walking on flat terrain, according to individual capabilities and clinical status.

The participants also underwent a psychological counselling programme (i.e. cognitive behavioural therapy strategies) consisting of two or three sessions per week of individual and/or group psychotherapy performed by clinical psychologists. When possible (1 day per week), additional sessions were conducted with the patients’ parents to improve their motivation for lifestyle change and interpersonal communication.

### 2.4 Experimental sessions

All procedures listed below were performed in the studied group of subjects (OB), over a 3‐day period, before (PRE) and after (POST) the 3‐week multidisciplinary BM reduction programme experiments. CTRL underwent only the PRE measurements. Experiments were carried out under continuous medical supervision and following standard safety procedures.

### 2.5 Anthropometric measurements

Appendicular (or limb) skeletal muscle mass (ASMM) was estimated non‐invasively by a bioelectrical impedance (BIA) (Human‐IM Scan; DS‐Medigroup, Milan, Italy) equation proposed by Kyle et al. (2003), which includes height, resistance, body mass, sex, age and reactance. The variables estimated by this equation have been shown to be strongly correlated with DEXA measurements, both in healthy and in diseased populations. Whole‐body resistance to an applied current (50 kHz, 0.8 mA) was measured with a tetrapolar device, with electrodes placed on the right wrist and ankle of the supine subjects lying comfortably in bed with limbs abducted from the body. To reduce measurement errors, special care was paid to the standardization of the variables known to affect measurement validity, reproducibility and precision. Measurements were performed according to the method of Lukaski (1987) (i.e. after 20 min of resting in a supine position with arms and legs relaxed and not in contact with other body parts) and in strictly controlled conditions. Fat‐free mass (FFM) was calculated with equations derived from a two‐compartment model (Gray et al., 1989). Fat mass (FM) was calculated as the difference between total BM and FFM; both variables were expressed as kilograms and as a percentage of BM (Table 1). The same investigators performed all examinations in PRE and in POST. Waist circumference was measured midway between the lower rib margin and the superior anterior iliac spine, by using a horizontally applied non‐stretch tape.

### 2.6 Blood samples

Blood samples were drawn from an antecubital vein of the arm by venipuncture at two time points: during the first visit at rest in the fasting state and 1 h before the PLM test (see above) about 2.5 h after a light meal. While endothelial and inflammation variables were determined during the second visit, blood chemistry variables were measured only during the first visit.

Blood for variables determined in plasma was collected in plain tubes containing EDTA, and then centrifuged at 653 *g* for 15 min at 4°C. Plasma samples were stored at −80°C until analysis. Blood for variables determined in serum was collected in plain tubes with a clotting activator and left to clot for a minimum of 30 min at room temperature, and then centrifuged at 1469 *g* for 10 min at 4°C. Serum was stored at −80°C until analysis.

### 2.7 Blood chemistry variables

Blood chemistry variables were determined by standard procedures (Rigamonti et al., 2020).

### 2.8 Proteomic analyses of biomarkers of endothelial function

Assessment of biomarkers of endothelial function was performed using a micro‐liquid chromatography–tandem mass spectrometry (microLC/MS‐MRM) method as described previously (Suraj et al., 2018, 2019; Walczak et al., 2015). The method was previously utilized to assess profiles of endothelial dysfunction accompanying impaired NO‐dependent vasodilation in atherosclerosis (Bar et al., 2019), high‐fat diet (Bar et al., 2020) or cancer (Suraj et al., 2019). The panel included protein and peptide biomarkers reflecting various aspects of *endothelial function* such as, for glycocalyx disruption: syndecan‐1 (SDC‐1); and for haemostasis: von Willebrand factor (vWF), tissue plasminogen activator (t‐PA), plasminogen activator inhibitor 1 (PAI‐1), thrombin activatable fibrinolysis inhibitor (TAFI), thrombospondin 1 (THBS‐1) and the soluble form of thrombomodulin (sTM). For variables evaluating *endothelial inflammation* we determined: soluble vascular cell adhesion molecule 1 (sVCAM‐1), soluble intercellular adhesion molecule 1 (sICAM‐1) and the soluble form of E‐selectin (sE‐sel). For variables evaluating *endothelial permeability* we determined: angiopoietin 1 (ANGPT‐1), angiopoietin 2 (ANGPT‐2), the soluble form of fms‐like tyrosine kinase 1 (sFLT‐1) and the soluble form of Tie‐2 receptor (sTie‐2). In terms of other proteins/peptides related to *vascular function* we determined: adrenomedullin (ADM), adiponectin (ADN), annexin V (ANXA5) and myelin‐associated glycoprotein (MAG).

A UFLC Nexera system (Shimadzu, Kyoto, Japan) connected to a highly sensitive mass spectrometer, QTrap 5500 (Sciex, Framingham, MA, USA), was used for targeted proteomic analyses. Briefly, during sample preparation plasma underwent proteolytic digestion using porcine trypsin to achieve unique and reproducible peptide sequences, applied as surrogates of the proteins suitable for microLC‐MS/MS analyses. A detailed description of the sample preparation protocol, as well as chromatographic and mass spectrometric parameters for the targeted analysis of selected biomarkers was presented elsewhere (Suraj et al., 2018, 2019).

### 2.9 Plasma nitrite (NO_2_−) and nitrate (NO_3_−) concentration

Nitrite (NO_2_^−^) and nitrate (NO_3_^−^) levels were measured by injecting 10 µL of extract into a dedicated HPLC system (ENO‐20, AMUZA, San Diego, CA, USA). In this system, NO_3_^−^ and NO_2_^−^ are first isolated from each other and from interfering substances on a separation column, NO_3_^−^ is reduced to NO_2_^−^ on a cadmium column, then both react with Griess reagent before being detected spectrophotometrically at 540 nm (Stamm et al., 2021). Plasma samples were precipitated with methanol in the ratio of 1:1 (v/v), and subsequent samples were centrifuged at 10,000 g for 10 min as described previously (Przyborowski et al., 2018).

### 2.10 Blood flow in the femoral artery during passive leg movement (PLM)

Blood flow measurements were performed at rest and during 1 min of PLM of the right leg, following standard guidelines (Gifford & Richardson, 2017). The subjects remained seated with their legs extended and supported for 15 min before data collection. Resting Doppler ultrasound (duplex mode, with simultaneous collection of vessel image with B‐mode and blood velocity with pulse‐wave mode) data were recorded for 60 s, and were followed by measurements performed during 60 s of cyclical passive knee extension and flexion movements. The movements were performed across a 90° range of motion (180°–90°–180°) at 1 Hz, following a metronome. The same trained researcher manually moved the subject's leg in PRE and POST. The subjects were instructed not to activate their muscles during the movements. The absence of active movements was ensured during preliminary practice runs. The same researcher performed the measurements in PRE and in POST.

Blood flow in the common femoral artery was estimated by measurements of blood flow velocity and vessel diameter distally to the inguinal ligament, 2–3 cm proximally to the bifurcation of the superficial and deep femoral artery, by using a Doppler ultrasound system (My Lab 25, Esaote) with a convex array transducer operating at the imaging frequency of 3.5 MHz. Measurements of the arterial lumen were made from B‐mode images in longitudinal view. Measurements of the vessel diameter were taken at the same time point in the cardiac cycle (peak of the R wave derived from the ECG). Blood flow velocities were collected with the sample volume covering more than 75% of the arterial lumen, and with the insonation angle always kept <60°. Arterial blood flow was manually calculated second by second by the same operator, by multiplying arterial cross‐sectional area and mean blood flow velocity. Measurements were performed in duplicate, and the protocol was repeated after 15 min of recovery. Measurements obtained during the two repetitions were superimposed, and average values were obtained for each subject and retained for data analysis. Resting blood flow and peak blood flow during PLM were calculated. The difference between peak and resting blood flow (Δpeak) was also calculated. The area under the blood flow versus time response (area under the curve, AUC) was obtained by calculating the integral of the function over the entire 60 s, after subtracting the resting baseline value (see Gifford & Richardson, 2017). Blood flow data were expressed in absolute values and were also normalized for 100 g of skeletal muscle tissue of the lower limb, determined by ASMM.

### 2.11 Incremental exercise test and systemic variables of oxidative function

The subjects performed an incremental exercise test (INCR) up to voluntary exhaustion on an electronically braked cycle ergometer (Corival cpet, Lode, the Netherlands) to determine peak pulmonary oxygen uptake (${\dot{V}}_{O_{2}\mathrm{peak}}$), the gas exchange threshold (GET) and the respiratory compensation point (RCP). The test started with 20 W for 2 min, and then a 20 W increase in work rate was imposed every minute until voluntary exhaustion. Pedalling frequency was digitally displayed to the subjects, who were asked to keep a constant cadence throughout the tests at their preferred value (between 60 and 80 rpm). Voluntary exhaustion was defined as the incapacity to maintain the imposed load and pedalling frequency despite vigorous encouragement by the researchers. Pulmonary ventilation (${\dot{V}}_{E}$), pulmonary oxygen uptake (${\dot{V}}_{O_{2}}$) and CO_2_ output (${\dot{V}}_{CO_{2}}$) were determined breath‐by‐breath by a metabolic cart (Ergostick; Geratherm Respiratory, Bad Kissingen, Germany). Expiratory flow measurements were performed by a turbine flow meter, calibrated before each experiment by a 3‐L syringe at different flow rates. ${\dot{V}}_{O_{2}}$ and ${\dot{V}}_{CO_{2}}$ were determined by continuously monitoring $P_{O_{2}}$ and $P_{CO_{2}}$ at the mouth throughout the respiratory cycle and from established mass balance equations. O_2_ and CO_2_ analysers were calibrated before each experiment by utilizing gas mixtures of known composition. Peak values of the main variables were taken as the highest 20‐s mean values attained before the subject's voluntary exhaustion. The gas exchange ratio (*R*) was calculated as ${\dot{V}}_{CO_{2}}$/${\dot{V}}_{O_{2}}$. GET and RCP were identified by standard criteria (Beaver et al., 1986; Wasserman & Whipp, 1975). HR was determined continuously by a chest band (Polar Electro, Oulu, Finland); mean values were calculated every 5 s.

### 2.12 Statistical analysis

Data are presented as means ± standard deviation (SD), with the exception of the second figure, where SE is used for clarity. Statistical significance of differences between the two groups (CTRL vs. OB) was checked by a two‐tailed Student's unpaired *t*‐test. Statistical significance of differences observed in the participants with obesity before (PRE) and after (POST) the multidisciplinary in‐hospital body mass reduction programme was also checked by a two‐tailed Student's paired *t*‐test. Analysis of covariance (ANCOVA) was performed to detect the influence of the covariate mean arterial pressure on differences observed for PLM variables. Linear regression and correlation analysis were carried out by using the least‐squared residuals method. The level of significance was set at 0.05. Outliers identification was performed by the ROUT method. Statistical analyses were done using a commercially available software package (Prism 8.0; GraphPad Software, San Diego, CA, USA).

## 3 RESULTS

Systolic and diastolic blood pressure at rest were slightly higher in OB versus CTRL (Table 1).

### 3.1 Anthropometric variables

Except for height, anthropometric variables (Table 1) determined in the patients with obesity (OB) significantly differed from those of the control group (CTRL). OB showed higher values of BM (+54 kg), absolute FFM (+12 kg) and fat mass (+42 kg), and higher appendicular skeletal muscle mass (ASMM) (+1.7 kg) compared to CTRL. When FFM and FM were expressed as percentages of body mass, OB showed a greater percentage of fat mass and a lower percentage of fat‐free mass. Waist circumference, taken as an estimate of visceral fat, was higher in OB versus CTRL (+38 cm).

In OB BM (by ∼5 kg, corresponding to ∼4% of the initial BM), BMI and FM (both as a percentage and kg) were significantly lower in POST than in PRE (*P *< 0.001). FFM (expressed as a percentage) was higher in POST versus PRE, whereas there was no change in FFM expressed in kg (*P *= 0.61). ASMM was about 3% lower in POST versus PRE.

### 3.2 Blood variables

Baseline blood chemistry variables are summarized in Table 2. Fasting insulin and HOMA‐IR were significantly higher, whereas HDL‐cholesterol was significantly lower, in OB versus CTRL (Table 2). In OB patients, several indices (Table 3) indicated endothelial dysfunction and impaired systemic NO bioavailability, including low plasma NO_2_^−^ levels and higher ANGPT‐2 and ANGPT‐2/ANGPT‐1 ratios in OB versus CTRL. In contrast, biomarkers of endothelial inflammation, such as serum soluble vascular cell adhesion molecule‐1 (sVCAM‐1), serum soluble intercellular adhesion molecule‐1 (sICAM‐1), soluble form of E‐selectin (sE‐sel) and interleukin‐6 (IL‐6) were not elevated in OB versus CTRL (Table 3). However, adrenomedullin (ADM) plasma concentration was lower, while myelin‐associated glycoprotein (MAG), thrombospondin 1 (THBS‐1) concentrations and the ANGPT‐2/ANGPT‐1 ratio were higher in OB versus CTRL (Table 3). Despite the notable differences in endothelial biomarker profile in OB versus CTRL, 3‐week multidisciplinary body mass did not significantly alter these biomarkers, but it did result in a significant reduction in sE‐sel and MAG (Table 3). Total cholesterol and low‐density lipoprotein (LDL)‐cholesterol were lower in POST versus PRE (Table 2). No significant differences were observed for the other variables.

**TABLE 2:** Blood chemistry variables in controls (CTRL), obese patients (OB) before (PRE) and following (POST) standard interventions.

	CTRL	OB–PRE	*P*	OB–POST	*P*
Fasting glucose (mmol/L)	4.66 ± 0.16	4.76 ± 0.25	0.35	4.6 ± 0.3	0.08
Fasting insulin (mU/L)	8.9 ± 3.3	18.7 ± 9.0	**0.01**	18.0 ± 7.2	0.63
HOMA‐IR	1.84 ± 0.67	4.0 ± 2.1	**0.01**	3.8 ± 1.7	0.48
Total cholesterol (mg/dL)	167.4 ± 17.2	158.5 ± 28.7	0.46	138.7 ± 16.7	**0.001**
LDL‐cholesterol (mg/dL)	107.7 ± 12.8	108.5 ± 26.0	0.94	89.8 ± 16.5	**<0.001**
HDL‐cholesterol (mg/dL)	53.43 ± 10.58	36.5 ± 8.1	**<0.001**	34.7 ± 7.8	0.06
Triglycerides (mg/dL)	68.0 ± 19.2	93.6 ± 45.2	0.17	93.8 ± 36.7	0.97
C‐reactive protein (mg/dL)	0.03 ± 0.05	0.20 ± 0.23	0.07	0.16 ± 0.23	0.21

Data are means ± SD. *P*‐values related to differences between CTRL and OB‐PRE were checked by means of Student's unpaired *t*‐test; *P*‐values related to differences between OB‐PRE and OB‐POST were checked by means of Student's paired *t*‐test. Values shown in bold indicate statistical significance. HDL, high‐density lipoprotein; HOMA‐IR, homeostasis model for insulin resistance; LDL, low‐density lipoprotein.

**TABLE 3:** Biomarkers of endothelial function, inflammation and permeability in controls (CTRL), obese patients (OB) before (PRE) and following (POST) standard interventions.

	CTRL	OB–PRE	*P*	OB‐POST	*P*	
Endothelial function						
[NO_2_^−^] (µmol/L)	0.51 ± 0.49	0.18 ± 0.09	**0.02**	0.13 ± 0.10	0.12	
[NO_3_^−^] (µmol/L)	26.7 ± 8.0	22.3 ± 7.5	0.16	19.7 ± 8.1	0.35	
[NO_2_^−^] + [NO_3_^−^] (µmol/L)	27.2 ± 8.2	22.3 ± 7.5	0.13	18.5 ± 9.3	0.19	
[ADM] (nM)	50.01 ± 19.65	36.70 ± 13.21	**0.05**	38.10 ± 13.08	0.72	
[ADN] (µM)	0.046 ± 0.034	0.039 ± 0.032	0.63	0.030 ± 0.29	0.05	
[ANXA5] (nM)	10.12 ± 6.54	7.82 ± 4.45	0.29	5.94 ± 4.55	0.28	
[MAG] (nM)	25.56 ± 13.74	57.02 ± 15.60	**<0.001**	43.56 ± 21.56	**0.03**	
[Syndecan‐1] (nM)	194.7 ± 36.1	173.1 ± 28.6	0.10	174.9 ± 33.4	0.86	
[vWF] (µM)	0.505 ± 0.197	0.386 ± 0.149	0.09	0.430 ± 0.252	0.44	
[t‐PA] (nM)	12.43 ± 5.40	11.37 ± 4.15	0.58	12.64 ± 5.40	0.50	
[PAI‐1] (µM)	0.278 ± 0.063	0.273 ± 0.060	0.82	0.259 ± 0.078	0.56	
[TAFI] (µM)	0.026 ± 0.019	0.037 ± 0.014	0.12	0.035 ± 0.026	0.64	
[THBS‐1] (µM)	1.987 ± 0.784	2.603 ± 0.501	**0.02**	2.365 ± 0.833	0.32	
[TM] (nM)	101.2 ± 30.81	119.6 ± 28.3	0.13	114.0 ± 16.1	0.35	
Endothelial inflammation						
[sVCAM‐1] (nM)	4.31 ± 3.84	3.80 ± 2.07	0.67	3.17 ± 1.58	0.31	
[sICAM‐1] (nM)	2.74 ± 2.10	3.01 ± 2.72	0.75	3.5 ± 1.8	0.38	
[sE‐sel] (nM)	11.72 ± 8.67	11.36 ± 5.41	0.90	4.72 ± 3.14	**0.004**	
Endothelial permeability						
[ANGPT‐1] (nM)	6.72 ± 3.75	5.18 ± 2.90	0.25	5.30 ± 3.71	0.91	
[ANGPT‐2] (nM)	16.24 ± 14.00	26.54 ± 8.20	**0.03**	20.82 ± 15.06	0.10	
ANGPT‐2/ANGPT‐1	3.30 ± 2.67	8.18 ± 8.60	**0.04**	4.14 ± 3.39	0.08	
[sFLT‐1] (nM)	0.22 ± 0.09	0.27 ± 0.70	0.13	0.24 ± 0.09	0.34	
[sTie‐2] (nM)	5.15 ± 2.10	4.80 ± 3.05	0.74	4.08 ± 3.47		0.53
Other markers						
IL‐6 (pg/mL)	1.6 ± 0.2	2.8 ± 1.9	0.07	2.8 ± 1.5		0.90
Alpha 1 glycoprotein (mg/mL)	65.0 ± 11.4	77.3 ± 19.6	0.12	73.5 ± 21.5		0.32
Testosterone (nmol/L)	18.2 ± 5.6	13.2 ± 7.1	0.10	18.1 ± 18.9		0.30
Cortisol (µg/dL)	13.1 ± 3.7	13.0 ± 4.6	0.94	13.4 ± 4.2	0.76	

Data are means ± SD. *P*‐values relate to differences between groups by means of Student's unpaired *t*‐test. Values shown in bold indicate statistical significance. [ADM], adrenomedullin; [ADN], adiponectin; [ANGPT‐1], angiopoietin 1; [ANGPT‐2], angiopoietin 2; [ANXA5], annexin 5; IL‐6, interleukin‐6; [MAG], myelin‐associated glycoprotein; [NO_2_^−^], plasma nitrite concentration; [NO_3_^−^] plasma nitrate concentration; [NO_2_^−^] + [NO_3_^−^], sum of plasma nitrite and nitrate; [PAI‐1], plasminogen activator inhibitor‐1; [sE‐sel], soluble form of E‐selectin; [sFLT‐1], soluble forms‐like tyrosine kinase‐1; [sICAM‐1], serum soluble intercellular adhesion molecule‐1; [sTie‐2], soluble form of the receptor for angiopoietin 1; [sVCAM‐1], serum soluble vascular cell adhesion molecule‐1; [Syndecan‐1], serum syndecan‐1 concentration; [TAFI], activation of thrombin activatable fibrinolysis inhibitor; [THBS‐1], thrombospondin 1; [TM], thrombomodulin; [t‐PA], tissue‐type plasminogen activator; [vWF], vonWillebrand factor.

### 3.3 Endothelial function assessed by the PLM test

**FIGURE 1:**
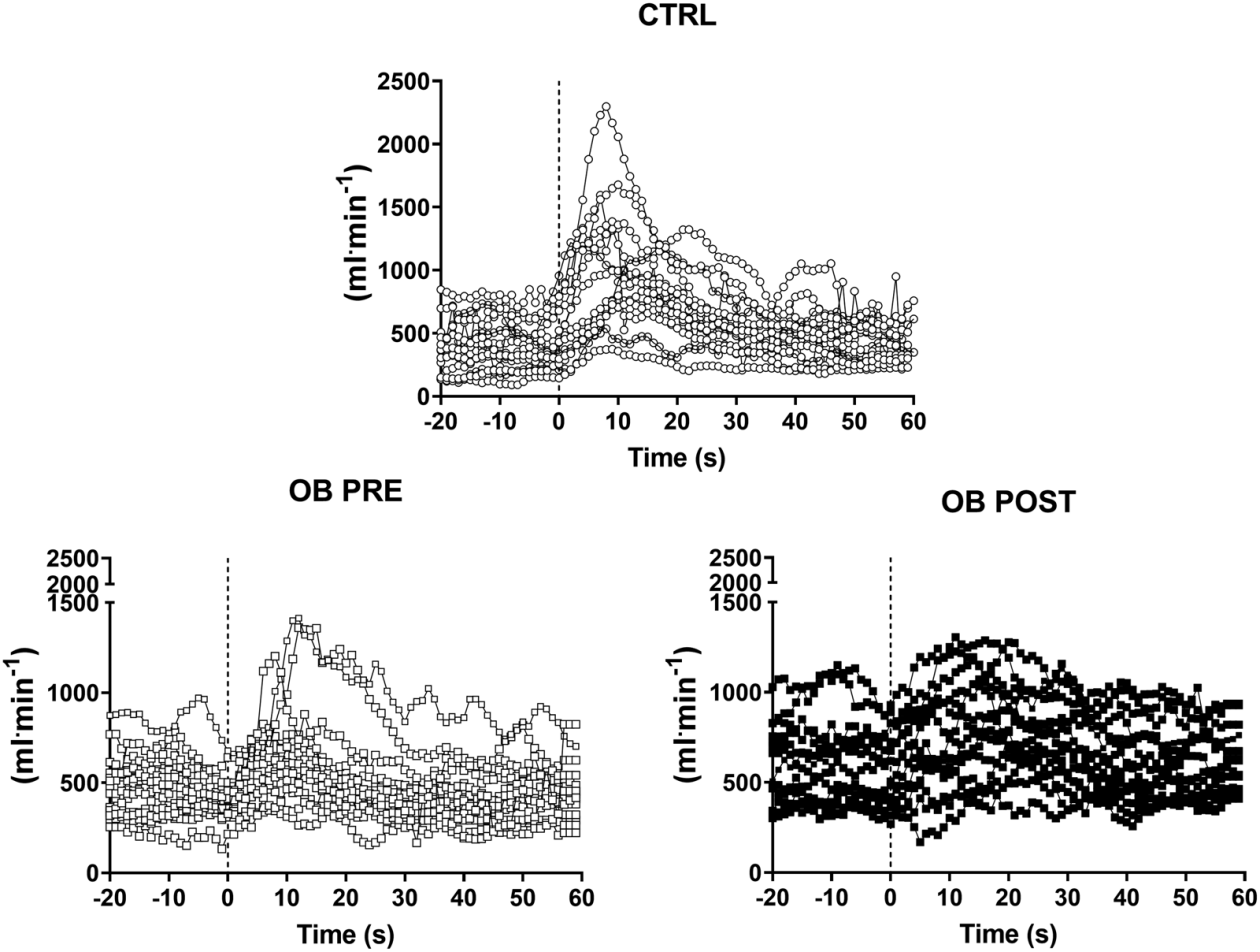
Individual values of blood flow in the common femoral artery at rest and in response to passive leg movement (PLM), in controls (CTRL), obese patients (OB) before (PRE) and after (POST) the standard 3‐week in‐hospital rehabilitation programme. The vertical line indicates the onset of PLM.

**FIGURE 2:**
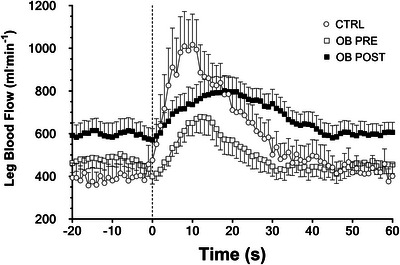
Mean (± SE) values of blood flow in the common femoral artery at rest and in response to passive leg movement (PLM), in controls (CTRL), obese patients (OB) before (PRE) and after (POST) the standard 3‐week in‐hospital rehabilitation programme. The vertical line indicates the onset of PLM.

**FIGURE 3:**
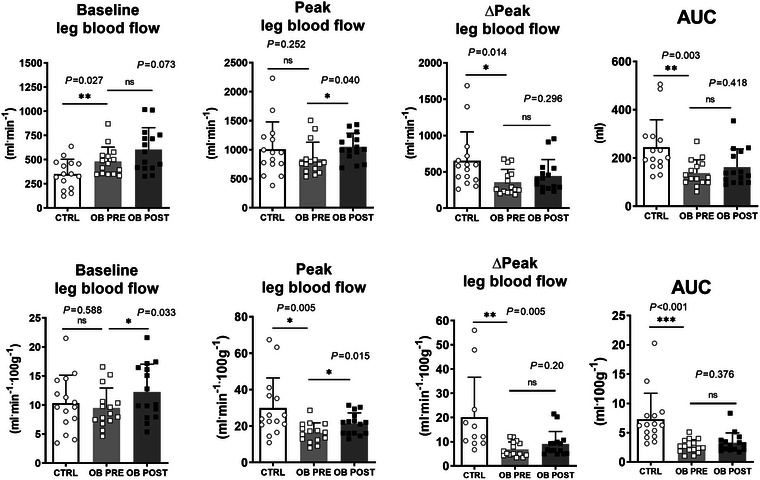
Mean (±SD) values of the PLM‐derived data in control participants (CTRL) and obese individuals (OB). ∆peak are changes in leg blood flow from baseline to peak blood flow and AUC is the area under the blood flow versus time curve. The upper graphs refer to absolute values whereas the lower graphs refer to data normalized by 100 g of leg lean mass.

Common femoral artery diameter was not different (*P *= 0.50) in CTRL (8.9 ± 1.6 mm) versus OB (8.4 ± 1.3). Individual leg blood flow values obtained at rest and during PLM are presented in Figure 1, and mean values for the three conditions are reported in Figure 2. Individual and mean values obtained in CTRL and in OB PRE and OB POST during the PLM test are shown in Figure 3. Baseline blood flow was higher in OB PRE than in CTRL (+31%; *P *= 0.03) (Figure 3). Leg blood flow increased very rapidly after the onset of the PLM in CTRL, reaching a peak after about 10 s. OB PRE showed a blunted response. Whereas peak blood flow was not different between groups (*P *= 0.25) (Figure 3), lower values were observed in OB PRE versus CTRL for ∆peak (−16%; *P *= 0.01) and for AUC (−43%; *P *= 0.003) (Figure 3).

Blood flow to a limb is related to limb muscle mass (Gifford & Richardson, 2017). Therefore, baseline, peak, Δpeak blood flows and AUC were also normalized for the estimated muscle mass of the limb, which was greater in OB (see ASMM values in Table 1). After this normalization, baseline blood flow was not different in OB PRE versus CTRL, whereas peak leg blood flow, ∆peak and AUC were significantly lower in OB PRE versus CTRL (−45%; *P *= 0.005, −65%; *P *= 0.002 and −63%; *P *< 0.001, respectively; Figure 3). ANCOVA analysis demonstrated that group differences in PLM variables were not driven by the small differences observed for the covariate mean arterial pressure.

In OB, the common femoral artery diameter was not different in POST versus PRE (*P *= 0.37). Individual and mean values of leg blood flow obtained at rest and during 1 min of PLM in OB POST are also presented in Figures 1 and 3. The blood flow *vs* time curves in OB POST were shifted upward compared to OB PRE. Figure 3 shows that baseline blood flow and peak blood flow were significantly higher in OB POST versus OB PRE, also after the data were normalized per muscle mass, whereas no significant differences were observed for Δpeak and AUC.

**FIGURE 4:**
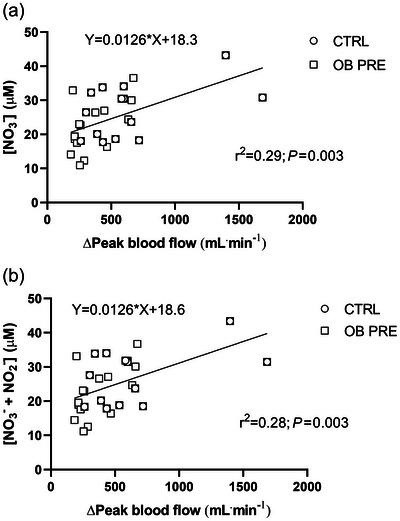
Individual values of [NO_3_^−^] (A) and the sum of [NO_3_^−^] and [NO_2_^−^] (B) as a function of ∆peak leg blood flow in obese (OB PRE) and control (CTRL) participants. [NO_3_^−^] and the sum of [NO_3_^−^] and [NO_2_^−^] are linearly correlated with ∆peak leg blood flow. See text for further details.

As illustrated in Figure 4, [NO_3_^−^] and the sum of [NO_2_^−^] and [NO_3_^−^] were significantly correlated with ∆peak blood flow (*P *= 0.003), although the *r* ^2^ values were rather low (*r* ^2^ = 0.29 and *r* ^2^ = 0.28, respectively). The equations describing the linear regression lines are also given in the figure. The two experimental points characterized by elevated Δpeak blood flow values were not outliers. This was demonstrated by the ROUT method, which we utilized by applying a *Q*‐value (maximal desired false recovery rate) of 1%. Moreover, after we excluded the two experimental data points from the correlation analyses, a statistically significant correlation was still present.

### 3.4 Incremental exercise and systemic variables of oxidative function

Values of the main respiratory, cardiovascular and metabolic variables obtained during the incremental exercise are shown in Table 4. Whereas ${\dot{V}}_{O_{2}\mathrm{peak}}$, ${\dot{V}}_{O_{2}}$ at GET and ${\dot{V}}_{O_{2}}$ at RCP, when expressed in absolute units, were not significantly different in OB PRE versus CTRL, when the variables were expressed per unit of BM the values were significantly (by about 40–50%) lower in OB PRE versus CTRL. ${\dot{V}}_{O_{2}\mathrm{peak}}$ values in OB PRE were typical for subjects with obesity (see e.g. Zuccarelli et al., 2021). OB and CTRL attained peak HR values corresponding to ∼84% and ∼90% of the age‐predicted maximum (calculated as 208 − 0.7 age; Tanaka et al., 2001), respectively. After considering also the *R* peak and RPE peak values, it can be assumed that exhaustion was substantially reached, although no validation test for the determination of maximal ${\dot{V}}_{O_{2}}$ (Poole & Jones, 2017) was carried out in the recovery phase. In any case, ${\dot{V}}_{O_{2}}$ at GET and at RCP represent variables that are not affected by the incremental test being truly maximal or not. In both OB PRE and CTRL, ${\dot{V}}_{O_{2}}$ at GET and at RCP occurred at about 60% and 85%–90% of ${\dot{V}}_{O_{2}\mathrm{peak}}$.

**TABLE 4:** Mean ± SD values of the main respiratory, cardiovascular and metabolic variables determined during the incremental exercise in obese patients (OB) before (PRE) and following (POST) standard interventions and in control participants (CTRL).

	CTRL	OB–PRE	*P*	OB–POST	*P*
Peak work rate (W)	236 ± 62	186 ± 36	**0.01**	196 ± 34	**0.003**
Peak work rate (W kg^−1^)	3.6 ± 1.0	1.6 ± 0.4	**<0.001**	1.7 ± 0.4	**<0.001**
${\dot{V}}_{O_{2}\mathrm{peak}}$ (L min^−1^)	2.870 ± 0.860	2.506 ± 0.360	0.15	2.604 ± 0.444	0.47
${\dot{V}}_{O_{2}\mathrm{peak}}$ (mL kg^−1^ min^−1^)	43.8 ± 14.5	21.3 ± 3.3	**<0.001**	23.1 ± 4.0	**0.01**
${\dot{V}}_{CO_{2}\mathrm{peak}}$ (L min^−1^)	3.461 ± 0.999	2.727 ± 0.466	**0.02**	2.880 ± 0.523	**0.03**
*R*_peak_	1.21 ± 0.09	1.09 ± 0.08	**<0.001**	1.11 ± 0.07	0.28
${\dot{V}}_{\mathrm{Epeak}}$ (L min^−1^)	114.0 ± 31.8	87.7 ± 10.7	**0.01**	100.1 ± 19.4	**0.01**
*V*_Tpeak_ (L)	2.6 ± 0.6	2.4 ± 0.5	0.36	2.5 ± 0.6	**0.04**
*f*_Rpeak_ (breaths min^−1^)	45 ± 8	38 ± 6	**0.02**	41 ± 7	0.10
$P_{\mathrm{ET}O_{2}\mathrm{peak}}$ (mmHg)	103 ± 9	94 ± 2	**0.001**	97 ± 3	**0.003**
$P_{\mathrm{ETC}O_{2}\mathrm{peak}}$ (mmHg)	37 ± 5	36 ± 2	0.89	34 ± 2	**0.002**
EqO_2_	39 ± 6	33 ± 3	**0.007**	37 ± 4	**0.004**
EqCO_2_	32 ± 4	31 ± 2	0.31	33 ± 2	**0.001**
${\dot{V}}_{O_{2}\mathrm{GET}}$ (L min^−1^)	1.721 ± 0.657	1.458 ± 0.296	0.18	1.576 ± 0.282	**0.04**
${\dot{V}}_{O_{2}\mathrm{GET}}$ (mL kg^−1^ min^−1^)	26.5 ± 10.7	12.3 ± 1.8	**<0.001**	13.9 ± 2.3	**0.001**
${\dot{V}}_{O_{2}\mathrm{GET}}$ (%${\dot{V}}_{O_{2}\mathrm{peak}}$)	62 ± 8	58 ± 6	0.28	60 ± 6	0.43
${\dot{V}}_{O_{2}\mathrm{RCP}}$ (L min^−1^)	2.478 ± 0.814	2.270 ± 0.281	0.37	2.329 ± 0.310	0.21
${\dot{V}}_{O_{2}\mathrm{RCP}}$ (mL kg^−1^ min^−1^)	37.8 ± 13.07	19.3 ± 3.1	**<0.001**	20.7 ± 3.3	**0.004**
${\dot{V}}_{O_{2}\mathrm{RCP}}$ (%${\dot{V}}_{O_{2}\mathrm{peak}}$)	86 ± 5	90 ± 6	**0.04**	88 ± 6	0.21
HR_peak_ (beats min^−1^)	177 ± 10	171 ± 12	0.13	172 ± 1	0.59
HR_peak_ (%HR_MAXpred_)	90 ± 6	84 ± 5	0.11	85 ± 4	0.29
RPE_peak_ (6–20)	19 ± 1	19 ± 1	0.35	19 ± 1	0.71

Data are means ± SD. *P‐*values releted to differences between CTRL and OB‐PRE were checked by means of Student's unpaired *t‐*test. *P*‐values related to differnces between OB‐PRE and OB‐POST were checked by means of Student's paired *t‐*test. Values shown in bold indicate statistical significance. EqCO_2_, ventilatory equivalents for CO_2_; EqO_2_, ventilatory equivalents for O_2_; *f*_R_, breathing frequency; HR, heart rate; HR_MAXpred,_ age‐predicted maximal heart rate; $P_{\mathrm{ETC}O_{2}}$, end‐tidal CO_2_ partial pressure; $P_{\mathrm{ET}O_{2}}$, end‐tidal O_2_ partial pressure; *R*, gas exchange ratio; RPE, rate of perceived exertion; ${\dot{V}}_{CO_{2}}$, carbon dioxide output; ${\dot{V}}_{E}$, pulmonary ventilation; ${\dot{V}}_{O_{2}}$, pulmonary oxygen uptake; ${\dot{V}}_{O_{2}\mathrm{GET}}$, pulmonary O_2_ uptake at GET; ${\dot{V}}_{O_{2}\mathrm{RCP}}$, pulmonary O_2_ uptake at RCP; *V*_T_, tidal volume.

No correlations were observed between individual values of ${\dot{V}}_{O_{2}\mathrm{peak}}$ (expressed in L min^−1^ or in mL kg^−1^ min^−1^) and peak blood flow, ∆peak and AUC (*P *> 0.05) obtained during the PLM test, either in OB or in CTRL.

All OB carried out the incremental exercise in POST except one, who did not complete the test for medical reasons and was excluded from the POST versus PRE comparison. Peak aerobic power and exercise tolerance improved in POST versus PRE. Peak work rate, ${\dot{V}}_{O_{2}\mathrm{peak}}$, ${\dot{V}}_{O_{2}}$ at GET and ${\dot{V}}_{O_{2}}$ at RCP were indeed significantly (by about 5–15%) higher in POST versus PRE.

## 4 DISCUSSION

An impaired endothelial function was observed in young individuals with obesity (OB) by utilizing the passive leg movement (PLM) test, which evaluates the blood flow increase in the common femoral artery during passive leg movements of a lower limb (Gifford & Richardson, 2017). Δpeak blood flow and AUC were significantly lower in OB PRE versus CTRL. Although peak blood flow was not different in the two groups, this observation could be explained by the higher baseline blood flow values in OB PRE, likely attributable to the greater appendicular muscle mass. After being normalized for the appendicular muscle mass, indeed, baseline blood flow values were not different in the two groups, and peak blood flow, Δpeak and AUC were significantly lower in OB PRE, supporting the concept of an impaired NO‐dependent vasodilator function. This impairment was associated with impairments of systemic variables evaluating oxidative metabolism during exercise, such as peak pulmonary O_2_ uptake (${\dot{V}}_{O_{2}\mathrm{peak}}$) and the two ‘ventilatory thresholds’ (GET and RCP), usually determined to evaluate the exercise tolerance of the subject. Thus, in young OB an impaired endothelial function can be added to the list of the previously described respiratory (LoMauro et al., 2016; Salvadego et al., 2015), cardiovascular (Ingul et al., 2010) and skeletal muscle (Lazzer et al., 2013; Salvadego et al., 2010) functional impairments.

To the best of our knowledge, this is the first study in which the PLM test has been utilized as a non‐invasive functional evaluation tool of endothelial function in patients with obesity. The hyperaemic response to PLM has been demonstrated to be mainly (although not exclusively, see e.g. Trinity et al., 2021) nitric oxide (NO)‐mediated (Mortensen et al., 2012; Trinity et al., 2012). During the PLM test, an increased shear stress on endothelial cells would induce an increased NO release, resulting in vasodilation of small feed arteries and microvascular beds (Gifford & Richardson, 2017). An attenuated hyperaemic response to the PLM test would then represent an impaired capacity by the vascular system to adjust the vascular tone to a given stimulus.

In the present study we observed lower levels of plasma nitrites (NO_2_^−^) in OB PRE versus CTRL, suggesting a lower systemic NO availability (sum of NO_2_^−^ and NO_3_^−^) in the OB PRE group. Both NO_2_^−^ and NO_3_^−^ can be reduced to NO in the so‐called nitrate–nitrite–NO pathway (Gladwin et al., 2005), especially in acidic/hypoxic conditions (Modin et al., 2001), to compensate for the lower NO bioavailability. The relevance of this mechanism in exercise conditions, however, needs further studies (Zoladz et al., 2024). In the present study we found a positive relationship between the hyperaemic response during PLM and NO bioavailability (sum of NO_2_^−^ and NO_3_^−^), both in CTRL and in OB PRE. This confirms that the hyperaemic response during PLM is mainly NO‐dependent, as previously demonstrated (Mortensen et al., 2012; Trinity et al., 2012). In a previous study by our group (Rasica et al., 2018), performed on adolescents with obesity, a short‐term (3 days) dietary supplementation with NO_3_^−^ increased plasma NO_3_^−^ levels and enhanced exercise tolerance (as demonstrated by the increased time to exhaustion during severe‐intensity exercise on a cycle ergometer), in association with a reduced O_2_ cost of exercise and a less pronounced amplitude of the slow component of the pulmonary ${\dot{V}}_{O_{2}}$ kinetics (Grassi et al., 2015). The studies suggest that in young individuals with obesity, NO bioavailability has a positive effect on exercise tolerance.

Endothelial dysfunction is often considered a harbinger of the development of insulin resistance (Buscemi et al., 2010), microvascular and macrovascular complications and cardiovascular diseases (Deanfield et al., 2007). These complications represent the main long‐term negative consequences of obesity, also in young patients. The availability of tools identifying and quantifying early signs of endothelial function impairment, before the onset of clinical complications, would have clinical relevance.

Other tests utilized for the assessment of endothelial function are invasive (e.g. the intra‐arterial infusion of the endothelium‐dependent vasodilator acetycholine [Mortensen et al., 2012], or the stimulation of β‐adrenergic‐mediated vasodilation [Limberg et al., 2022]) or more technically challenging (such as the flow‐mediated vasodilation test, FMD) (Harris et al., 2010) compared to the PLM test, which has proven to be relatively simple to perform and reliable (Gifford & Richardson, 2017). Moreover, as mentioned in the Introduction, the FMD and other invasive tests yielded conflicting results in patients with diabetes, particularly of young age. In the present study, OB PRE presented signs of dyslipidaemia (lower HDL‐cholesterol) and insulin resistance (higher fasting insulin and HOMA‐IR), confirming the presence of an increased risk of diabetes and cardiovascular diseases. However, no signs of diabetes (normal fasting glucose levels), hypertension or cardiovascular diseases were present. This suggests that young OB individuals exhibit endothelial dysfunction, which was confirmed by the PLM test and by observation of low NO_2_^−^ plasma levels. These data indicate a risk for diabetes, microvascular and macrovascular complications, and cardiovascular diseases in these patients, which can be identified before these issues become clinically apparent. This underscores the potential relevance of the PLM test and plasma NO_2_^−^ level as screening tools.

On the other hand, whereas for peripheral vascular function assessed by FMD a capacity to predict the incidence of subsequent cardiovascular diseases has been demonstrated (Yeboah et al., 2009), to the best of our knowledge no such inferences have so far been demonstrated for PLM data. Further research is needed on this topic. The inferences, however, seem highly likely after considering the strong pathophysiological connections between endothelial dysfunction and the progression of cardiovascular and metabolic diseases.

Interestingly, young OB who displayed impaired NO‐dependent function, as determined by PLM and lower plasma NO_2_^−^ levels, did not exhibit elevations in typical biomarkers of endothelial inflammation or haemostasis commonly associated with atherosclerosis (Bar et al., 2019; Walczak et al., 2015). However, the elevated ANGPT‐2 levels and the increased ANGPT‐2/ANGPT‐1 ratio were consistent with early alterations in endothelial permeability, which may serve as one of the most sensitive read‐outs of endothelial dysfunction (Bar et al., 2019). Additionally, while ADM levels were reduced, THBS1 concentrations were elevated. Elevated ADM levels have recently been linked to insulin resistance, and both endothelial loss and blockade of the ADM receptor have been shown to improve obesity‐induced insulin resistance (Cho et al., 2025), suggesting that the low ADM observed in young OB patients may represent a compensatory response of the early phase of the disease. However, ADM has also been shown to have vasoprotective effects (Liu et al., 2024), including increasing NO levels and exerting anti‐inflammatory and anti‐oxidant effects via the receptor–Akt pathway. THBS1, which was elevated in our study, has previously been identified as a sensitive predictor of non‐alcoholic fatty liver disease (NAFLD) in young obese individuals (Obradovic et al., 2021) and has been linked to the pathophysiology of obesity, diabetes and cardiovascular diseases (Gutierrez & Gutierrez, 2021). Thus, the endothelial dysfunction endotype in young obese individuals of this study was characterized by impaired PLM, low plasma NO_2_^−^ levels, an elevated ANGPT‐2/ANGPT‐1 ratio, and reduced ADM and elevated THBS1. These findings are consistent with our previous studies in mice, which demonstrated a disease‐specific profile of systemic biomarkers of endothelial dysfunction, despite similar impairment of NO‐dependent function in atherosclerosis (Bar et al., 2019) and cancer (Suraj et al., 2019) models of mice fed a high‐fat diet (Bar et al., 2020). Collectively, these results support the concept of the insidious nature of endothelial dysfunction in young OB, which manifests at the level of NO‐dependent function but does not correspond to distinct changes in classical blood biomarkers of endothelial dysfunction. On the other hand, these findings suggest that, for detecting endothelial dysfunction in young OB, the ANGPT‐2/ANGPT‐1 ratio, ADM and THBS1 should be considered as additional valuable biomarkers for assessing the endotype of endothelial dysfunction in young individuals with obesity with impaired NO‐dependent function. However, the limitation of this assumption is that the 3‐week body mass reduction intervention did not result in changes in plasma NO_2_^−^ levels or any of the biomarkers identified in OB versus CTRL. Similarly, no significant changes in the ANGPT‐2/ANGPT‐1 ratio were observed after a 3‐week multidisciplinary weight reduction programme in young OB (Table 3). This suggests that a longer weight reduction period is necessary to detect a clear improvement in endothelial function, as assessed by the biochemical markers studied. Further studies with longer interventions are needed to confirm this hypothesis.

On the functional levels, in the present study, the 3‐week body mass reduction intervention partially improved the PLM test, suggesting an effect on endothelial function. The multidisciplinary programme lowered body mass, BMI, total cholesterol and LDL‐cholesterol, and increased peak work rate, ${\dot{V}}_{O_{2}\mathrm{peak}}$, ${\dot{V}}_{O_{2}}$ at GET and ${\dot{V}}_{O_{2}}$ at RCP (when the last three variables were expressed as mL kg^−1^ min^−1^). For the PLM test an upward‐shifted blood flow versus time curve was observed in OB POST versus OB PRE. Baseline and peak leg blood flow normalized for leg skeletal muscle mass were higher in OB POST versus OB PRE, with no change in ∆peak.

The partial improvement of endothelial function observed in OB POST versus OB PRE in the present study confirms the reversibility of endothelial dysfunction found in other studies (Kerr et al., 2011; MacInnis & Gibala, 2017; Sciacqua et al., 2003; Watts et al., 2004; Wong et al., 2018) following exercise and dietary interventions of longer duration (from 6 weeks to 2 years), in which FMD was utilized. On the contrary, Clifton et al. (2005) did not report any improvement in the FMD responses after 3 months of body mass loss intervention.

The present study has limitations. The short duration (3 weeks) of the body mass reduction intervention may have limited its effectiveness in improving endothelial function. Future studies should evaluate longer interventions. No supramaximal validation test could be performed to strengthen the confidence in the ${\dot{V}}_{O_{2}\mathrm{peak}}$ measurements as estimates of ${\dot{V}}_{O_{2}\max}$ (Poole & Jones, 2017). Only male patients were investigated, limiting the generalizability of the findings. Previous evidence indicates sex‐specific differences in vascular pathophysiology, with females exhibiting greater susceptibility to diet‐induced endothelial stiffening (Padilla et al., 2019) and distinct mechanisms underlying microvascular dysfunction (Davel et al., 2018). Therefore, validation of the results of the present study in female cohorts is warranted. As pointed out by Gifford and Richardson (2017), as with all bioassays of vascular function there is an appreciable amount of variability with the PLM response, with a ‘within‐day and between‐day coefficient of variation of 15–20%’. Also considering this variability, in the present study PLM measurements were performed in duplicate, and average values were calculated and retained for the analysis. In any case, as shown in Figure 7 of the above‐mentioned paper (Gifford & Richardson, 2017), the effect of the experimental condition on PLM‐induced hyperaemia generally substantially outweighs the level of random variation in the response. Nonetheless, we recognize that the lack of repeated measurements in the control group represents another limitation of the study.

In conclusion, our study revealed an impaired endothelial function in young individuals with obesity, as detected by the simple method evaluating the hyperaemic response in the common femoral artery during PLM, as determined by Doppler ultrasound. The impaired endothelial function was associated with impairments of peak oxidative metabolism and exercise tolerance. The PLM test could be of interest as a screening tool, since the impaired endothelial function was detected in young obese patients in whom the clinical complications deriving from insulin resistance, microvascular and macrovascular impairments were still not present. Blood biomarkers of endothelial barrier function and permeability were more sensitive in detecting the early endothelial responses to body mass reduction than other blood variables. A short (3 weeks) period of rehabilitation, including exercise, diet and psychological counselling, partially corrected the observed impairments.

## AUTHOR CONTRIBUTIONS

Lucrezia Zuccarelli, Alessandro Sartorio, and Bruno Grassi conceived and designed research; Lucrezia Zuccarelli, Giovanni Baldassarre, Adele Bondesan, Diana Caroli, Roberta DeMicheli, Gabriella Tringali, and Thomas Favaretto performed experiments; Lucrezia Zuccarelli, Giovanni Baldassarre, Adele Bondesan, Diana Caroli, Roberta DeMicheli, Gabriella Tringali, Thomas Favaretto, Joanna Suraj‐Prazmowska, Anna Kurpinska, Joanna Majerczak and Stefan Chlopicki analysed data; Lucrezia Zuccarelli, Giovanni Baldassarre, Adele Bondesan, Diana Caroli, Roberta DeMicheli, Gabriella Tringali, Thomas Favaretto, Joanna Suraj‐Prazmowska, Anna Kurpinska, Joanna Majerczak, Stefan Chlopicki, Jerzy A. Zoladz, Alessandro Sartorio and Bruno Grassi interpreted results of experiments; Lucrezia Zuccarelli prepared figures; Lucrezia Zuccarelli, Joanna Suraj‐Prazmowska, Anna Kurpinska, Joanna Majerczak, Stefan Chlopicki, Jerzy A. Zoladz, Alessandro Sartorio and Bruno Grassi drafted manuscript; Lucrezia Zuccarelli, Giovanni Baldassarre, Joanna Suraj‐Prazmowska, Anna Kurpinska, Joanna Majerczak, Stefan Chlopicki, Jerzy A. Zoladz, Alessandro Sartorio and Bruno Grassi edited and revised manuscript. All authors have read and approved the final version of this manuscript and agree to be accountable for all aspects of the work in ensuring that questions related to the accuracy or integrity of any part of the work are appropriately investigated and resolved. All persons designated as authors qualify for authorship, and all those who qualify for authorship are listed.

## CONFLICT OF INTEREST

No conflicts of interest, financial or otherwise, are declared by the authors. The results of the study are presented clearly, honestly, and without fabrication, falsification, or inappropriate data manipulation.

## Data Availability

Source data for this study are not publicly available due to privacy or ethical restrictions. The source data are available to verified researchers upon request by contacting the corresponding author.
